# Prevalence and Determinants of Undernutrition in A Sample of Dutch Community-Dwelling Older Adults: Results from Two Online Screening Tools

**DOI:** 10.3390/ijerph16091562

**Published:** 2019-05-04

**Authors:** Jos W. Borkent, Elke Naumann, Emmelyne Vasse, Ellen van der Heijden, Marian A. E. de van der Schueren

**Affiliations:** 1 Dutch Malnutrition Steering Group, Nicolaas Witsenkade 13hs, 1017 ZR Amsterdam, The Netherlands; jos.borkent@han.nl (J.W.B.); e.naumann@han.nl (E.N.); e.vasse@stuurgroepondervoeding.nl (E.V.); stuurgroep@stuurgroepondervoeding.nl (E.v.d.H.); 2 Faculty of Health and Social Studies, Department of Nutrition and Health, HAN University of Applied Sciences, Kapittelweg 33, 6525 EN Nijmegen, The Netherlands; 3 Department of Dietetics, Hospital Gelderse Vallei, Willy Brandtlaan 10, 6716 RP Ede, The Netherlands; 4 Department of Nutrition and Dietetics, Amsterdam UMC, Vrije Universiteit, De Boelelaan 1117; 1081 HV Amsterdam, The Netherlands

**Keywords:** undernutrition, malnutrition, community-dwelling older adults, nutritional risk, self-screening, screening tools, risk screening

## Abstract

To stimulate undernutrition screening among Dutch community-dwelling adults, a website was developed with general information on healthy eating for healthy aging and self-tests. Based on cross-sectional data obtained from the self-tests, we studied nutritional risk factors (early determinants) as well as risk of undernutrition (late symptoms). SCREEN II (*n* = 2470) was used to asses nutritional risk factors. This tool consists of 16 items regarding nutritional intake, perception of body weight, appetite, oral health and meal preparation. An adjusted SNAQ65+ (*n* = 687) was used to assess risk of undernutrition. This four-item tool contains questions on weight loss, appetite, walking stairs and body mass index. Differences between age-groups (65–74, 75–84, ≥85) were tested by logistic regression. Overall prevalence of nutritional risk factors was 84.1%, and increased risk of undernutrition was 56.8%. Participants aged ≥85 scored worst on almost all items of the SCREEN II and the SNAQ65+. In conclusion: A large proportion of older adults reported early determinants for increased nutrition risk, while a smaller, yet remarkable proportion scored positive on undernutrition risk. Internet screening may be a useful, contemporary, and easy, accessible way to reach older adults who are at nutritional risk and may thus contribute to early identification and prevention of undernutrition.

## 1. Introduction

In the Netherlands, undernutrition rates in community-dwelling older adults range from 10% to 35%, depending on level of care and age [1,2,3]. While these rates are lower compared to hospitals and nursing homes, in absolute numbers, the largest number of undernourished older adults live at home [4]. Undernutrition is associated with adverse outcomes such as impaired recovery from diseases, cognitive decline, institutionalization, and mortality [5,6,7]. Therefore, early identification of older adults at nutritional risk is necessary to be able to take preventive measures. 

In the process of identifying persons at risk of undernutrition, screening tools are essential. Over the last decades, many screening tools have been developed and validated [8]. Most screening tools for undernutrition in older adults include low body mass index (BMI), loss of (muscle) mass, and/or impaired functioning as criteria [9]. However, these phenotypic, late symptoms of undernutrition indicate that a person is already at high risk or even undernourished [10]. Undernutrition should preferably be prevented in an earlier stage [11]. The preceding stages of undernutrition are characterized by the presence of early determinants such as problems with poor appetite, low food intake or difficulties with meal preparation [11]. Most screening tools only briefly address these early determinants. 

In the Netherlands, screening for undernutrition in the community is mainly done by general practitioners (GPs), nurse practitioners, and home care nurses [12]. However, not all older adults attend GP offices regularly, not all older adults who visit a general practitioner are screened for undernutrition, and not all older adults receive home care. Therefore, a large group of older adults may be at risk for undernutrition without being identified. E-health initiatives offer new possibilities for self-screening; in the Netherlands, internet access of adults aged >65 year is 86.4%, and over half (52.5%) of older adults use internet to search for health information [13]. 

In 2017, the Dutch Malnutrition Steering Group, with financial help from the Dutch government, launched a website with general information on healthy eating for healthy aging and self-tests. On this website www.goedgevoedouderworden.nl—translated as healthy eating for healthy aging—older adults or their informal caregivers can test their nutritional risk by answering questions on early determinants of undernutrition (based on the validated screening tool ‘Seniors in the Community: Risk evaluation for eating and nutrition, Version II’ (SCREEN II)) [14]. They can also test their undernutrition risk by answering questions on late symptoms of undernutrition (based on the modified version of ‘Short Nutritional Assessment Questionnaire for 65+’ (SNAQ^65+^)) [15]. After filling out the test for early determinants (SCREEN II), participants receive personalized feedback and advice based on their answers to each question. For example, if the outcome of the test shows problems with preparing meals, advice will be shown for this problem. If someone is found at risk for undernutrition or at high nutritional risk, the advice is to visit the GP or a dietitian. 

Previous studies among Dutch older adults were mostly based on undernutrition screening of late symptoms (weight loss, BMI, functionality) [1,2,3]. From previous research, we know that aging is a risk factor for late symptoms of undernutrition [16]. Very few data on prevalence of early determinants (nutritional risk factors) of undernutrition are available [17]. Data for early determinants in relation to aging are lacking. Therefore, we explored differences in both early determinants and late symptoms of undernutrition between age-groups of Dutch community-dwelling older adults based on the cross-sectional data obtained from internet-based self-tests.

## 2. Materials and Methods

### 2.1. Participants

Data were obtained from the website www.goedgevoedouderworden.nl. No recruitment was performed; data were used from attenders from the website that filled in the self-tests. All data were obtained anonymously. Only the age, gender, and zip code of the participants were stored in each self-test. Data-collection was done in the period April 2017–February 2019.

For all self-tests, the following inclusion criteria were used: Self-test had to be completed fully, and participant was aged >65 year. 

### 2.2. Measurements

The website provides two tools for assessing undernutrition risk: SCREEN II, a tool to assess nutritional risk, and a modified version of SNAQ^65+,^ a tool to assess undernutrition risk. Both tools could be filled out by the participant or by his/her informal care giver.

*SCREEN II:* The SCREEN II has been validated in community-dwelling older adults aged >65 year in Canada and New Zealand and can be self-administered. In these validation studies, high agreement was seen between SCREEN II and a nutritional risk assessment by a dietitian [14,18]. SCREEN II is a 16-item tool that covers nutritional risk factors such as weight change, perception of body weight, skipping meals, avoidance of products, appetite, intake of dairy/meat (replacements)/fruit and vegetables and fluids, problems with biting and chewing, use of meal replacements, eating together, meal preparation, and problems with doing groceries. For each separate item, a score can be achieved ranging from 0–4 points, whereby a score of ≤2 points is categorized as a nutritional risk for that specific item [14]. The maximum score of the SCREEN II is 64 points. Based on Keller et al. [14], a score below 54 indicates an increased overall nutritional risk.

*The modified version of SNAQ^65+^:* SNAQ^65+^ has been validated in Dutch community-dwelling older adults and was validated as a predictive tool for 15 years mortality. The tool consists of 4 questions: Mid-upper arm circumference (<25 cm), weight loss (≥4kg last six months), appetite, and walking stairs [15]. Based on a pilot study (results not published), self-assessment of mid-upper-arm circumference showed to be unreliable. Therefore, for the purpose of online self-testing, mid-upper-arm circumference was replaced by BMI, as BMI correlates highly with mid-upper arm circumference [19]. BMI was categorized according to the recently published Global Leadership Initiative on Malnutrition consensus criteria for undernutrition (GLIM criteria) with the following cut-off points for low BMI: <20 kg/m^2^ for adults 65–70 years and <22 kg/m^2^ for adults ≥70 years [10]. 

According to SNAQ^65+^, participants were categorized at high risk for undernutrition if they scored BMI <20 or weight loss ≥4kg last six months; participants were categorized as moderate risk if they had low appetite and problems with walking stairs. On the website, participants who had lost ≥4 kg over the last six months were not further questioned on appetite, problems with walking stairs or BMI, as they had already scored high-risk.

### 2.3. Analyses

Data were checked for normality using a QQ-plot. Descriptive statistics were performed by reporting means with standard deviation and numbers with percentages for categorical data. In order to investigate which risk factors occurred most frequently, all items of SCREEN II were reported separately stratified by age-group (65–74, 75–84, ≥85). Differences between age-groups on separate items of SCREEN II were tested using a chi-square test. Based on logistic regressions, odds ratios with 95% CI were calculated for proportions reaching <54 points. Differences in total points between age-groups were tested using linear regression. Analyses were tested for confounding by gender; however, the effect size differed <10%, so no adjustments were made.

Differences between age-groups in outcome based on the modified version of SNAQ^65+^ were tested by logistic regression. To do so, the modified version of SNAQ^65+^ was dichotomized into low vs. moderate/high risk as well into low/moderate vs. high risk. As all data were anonymous, data of the two self-tests could not be analyzed at individual participant level. All analyses were performed in SPSS 24 (IBM, Chicago VS), and a *p*-value of <0.05 was considered significant.

## 3. Results

### 3.1. Screen II

In total, 2470 participants completed the SCREEN II questionnaire. The mean age of all participants was 74.3 (SD:6.9) years, and 57.8% of the participants were aged 65–74 years. The majority of the participants were female (75.5%). A minor part of the questionnaires was filled in by an informal caregiver (14.7%); this was highest in the age-group ≥85 years (58.3%).

The mean score was 47.8 (SD:7.1) in participants aged 65–74 years, 45.1 (SD:8.7) in the age-group 75–84 years, and 39.2 (SD:9.0) in participants aged ≥85 years. Proportion at risk (<54 points) was 81.4% in participants aged 65–74 years, 85.5% in age-group 75–84 years, and 95.8% in age-group ≥85 years. Differences between age-groups were significant (*p* < 0.05) for mean total scores as well for proportions <54 points (Table 1). 

In Table 2, nutritional risk factors based on SCREEN II are shown. Most frequently scored risk factors on SCREEN II were: Problems with perception of own weight (62.6%), a low intake of fruit and vegetables (67.5%)/meat (replacements) (55.4%)/dairy products (55.3%), limiting and avoiding products (40.6%), eating meals alone (40.7%) problems with preparing meals (39.5%) or changes in body weight (38.7%). Less frequently scored risk factors were the use of meal replacements (9.4%), unintentional weight loss (9.8%), problems with biting and chewing (14.9%) or coughing/pain when swallowing (17.5%), doing groceries (17.4%), skipping meals (21.5%), problems with appetite (24.1%) or low fluid intake (24.9%).

### 3.2. Adjusted SNAQ^65+^

The adjusted SNAQ^65+^ was filled out by 687 participants, mean age 77.6 years (SD: 8.4), and most of them were female (75.4%). Nearly half of the questionnaires were filled out by an informal caregiver (46.3%); this was highest in the age-group ≥85 years (76.3%). In total, 390 (56.8%) participants were at high risk for undernutrition and 6.0% at moderate risk (Table 3). Within the high-risk group, 60.0% had lost over 4 kg body weight in the last six months, and 40% had a low BMI. High risk on undernutrition was highest in the age-group ≥85; this group had 3.0 [95%CI: 1.9–4.4] times higher odds of being at high risk of undernutrition compared to participants aged 65–74 years. The higher odds were also seen in the comparison of low vs. moderate/high; participants aged ≥85 had 3.8 [95%CI: 2.6–6.0] times higher odds compared to participants aged 65–74 (Table 3 and Table 4).

## 4. Discussion

The results of two online self-screening tests for risk of undernutrition show different prevalence rates for early determinants of undernutrition (84.1%) vs. late symptoms of undernutrition risk (56.8%) in a sample of Dutch community-dwelling older adults. These findings underline our assumption that early identification, based on nutritional risk factors, may be helpful in undertaking preventive measures. Based on the 16 individual risk items of the SCREEN II, tailored individual advice can be given. At a group level, interventions should focus on risk factors that are most common. Our results also indicate the need for self-screening among community-dwelling older adults. More than 2000 valid screening tests were filled out within two years. A large proportion of the visitors of the website www.goedgevoedouderworden.nl was at risk for undernutrition (based on the adjusted SNAQ^65+^), and most visitors had many nutritional risk factors (based on SCREEN II). Proportions of visitors at risk for undernutrition and with nutritional risk factors increased with age; in participants aged ≥85, over half of the participants were at high risk for undernutrition, and nearly everyone (>95%) reported one or more nutritional risk factors. 

The most frequently reported nutritional risk factor in the older Dutch population was perception of body weight; 62.6% judged their body weight as too high or low (without distinguishing between the two). Both overweight and underweight may lead to undernutrition. Not only underweight or overweight is a risk factor for older adults, but also attempts to lose weight may lead to loss of muscle mass if protein intake is not sufficient. Weight loss in older adults is associated with loss of muscle and bone mass [20]. Therefore, attempts to lose weight should incorporate exercise and optimal protein intake in order to prevent older adults from losing muscle and bone mass [20]. Any unintentional weight loss should lead to further nutritional and physical assessment, no matter the BMI. 

Other frequently reported risk factors were a low intake of meat (replacements) or fish and low dairy intake. These products are a major source of protein in community-dwelling older adults [21,22]. An adequate protein intake is needed to maintain and restore muscle mass. Based on recent guidelines, an intake of >1.0 gram per kilogram body weight is advised for healthy older adults [23]. In addition to the higher daily recommendation, an even distribution of protein intake over the day is also important. An intake of >25 grams protein per meal could optimize synthesis of muscle mass [24]. Especially during breakfast and lunch, protein intake is known to be below this recommendation [21,25]. The low intake of meat (replacements), fish, and dairy products is therefore a risk factor for muscle loss. 

A large part of the participants frequently ate their meals alone, which could have a negative impact on food intake. When eating alone, people tend to eat less [26], food is rated less tasteful [27], and meals are skipped more frequently [28]. To improve food intake, focus should not only be on meal composition but also on the setting. For community-dwelling older adults, it is important to activate their social network in order to prevent them from eating alone. 

In our study, we found that nutritional risk factors as well as high risk for undernutrition were associated with age. This is in line with most other studies where higher age is associated with increased risk of undernutrition [29,30]. However, previous international studies based on SCREEN II showed no association with age or a higher risk in lower age-groups [31,32,33]. There are several reasons that could explain the differences. Previous studies tested the association for age based on a linear relationship, while in our study, age showed to have an exponential relationship. Further, especially participants above 85 years of age were at risk, and most previous studies had few participants in this age category. Last, we were not able to adjust for important confounders in our study such as marital status, education level, and physical activity levels, as these data were not available. Based on the strength of the association, it is not likely that confounding alone could explain the difference between age-groups; however, attenuation of results for the oldest age-group is expected. 

The proportions at risk for undernutrition based on SNAQ^65+^ and nutritional risk factors based on SCREEN II are higher in our study compared to previous Dutch studies. In these studies, prevalence rates for undernutrition based on SNAQ^65+^ differed between 10% and 35% [1,2,3] compared to 56.8% in our study. A similar difference is seen on nutritional risk factors based on SCREEN II; in a small, exploratory study by Haakma et al. [17], 67% was at risk compared to 84.1% in our population. This study was hampered by a low number of participants (*n* = 335), and only adults aged 75–85 were included. Not only Dutch studies showed lower prevalence rates on SCREEN II. Studies from Canada and New Zealand showed prevalence rates of 34–40% based on SCREEN II [31,32,33]. The higher risk on both tools in our study can be explained by the origin of our data; www.goedgevoedouderworden.nl was launched with the aim to raise attention to undernutrition and GPs and dietitians refer to the website. Visitors of the website may therefore have been less healthy or at suspected nutritional risk in comparison to a more general population. The high prevalence of undernutrition and nutritional risk factors underline the importance of a website that provides self-screening and information about (under)nutrition for community-dwelling older adults. 

As data were collected anonymously, we do not know whether the collected data on the adjusted SNAQ^65+^ and SCREEN II were based on partly the same, or a different sample of participants. Nevertheless, early determinants of undernutrition (SCREEN II) seemed to be more prevalent compared to late symptoms of undernutrition (SNAQ^65+^). It is important, and likely easier, to intervene on early determinants, because they develop into late symptoms such as loss of weight and muscle mass [11]. A website such as www.goedgevoedouderworden.nl can be a useful, contemporary tool, as it provides both self-tests as direct feedback how to improve the diet, based on nutritional risk factors. 

The adjusted version of SNAQ^65+^ used BMI instead of mid-arm circumference, as arm-circumference was hard for older adults to measure. However, also the use of self-reported data for BMI could be unreliable. Previous studies showed an underestimation of weight and overestimation of height in older adults resulting in a too-low BMI [34,35,36]. However, underestimation of BMI is most frequently seen in overweight and obese older adults [35,37] and less frequently in participants with normal or underweight [38]. More research is needed to study the value of self-reported data of anthropometric measurements. 

A recent study of O’Keeffe et al. [39] categorized determinants of undernutrition in seven domains. SCREEN II focuses mainly on food intake by addressing the oral, psychosocial, and nutritional domains, but not the medication and care, health, physical functioning, and lifestyle domain. Especially health-related problems are of interest as, next to nutrient intake, decreased absorption or an increased protein/energy need could result in undernutrition [40]. It is not known from our data how these domains would have affected outcomes on nutritional risk and undernutrition risk. 

Despite the high access to internet of older adults in the Netherlands (86.4%) [13], a minor group is not able to use the internet. Lower internet use is mainly seen in higher age-groups, females, persons living alone [41], and those with lower education levels [42]. These groups could therefore be underrepresented in our study. However, most of the participants in our study were female, and the oldest age-group was relatively well represented as their informal care givers filled in the questionnaires. Even though the number of people not having access to internet declines year by year [43], and even though persons with low internet literacy tend to ask their surroundings for help [41], it must be acknowledged that e-health initiatives might not reach the most vulnerable population. Nevertheless, e-health is regarded as a novel way of reaching out to the Dutch (older) population while internet access rates are rather high.

A strong point of our study is the large study sample with a large subgroup of participants aged ≥85 years. Therefore, we had enough power to show differences in prevalence rates as well as differences in subitems of both screening tools across different age-groups. 

Finally, some limitations should be considered. First, the use of self-reported data could have led to misclassification. Secondly, the adjusted SNAQ^65+^ is not a validated tool, as it replaces arm-circumference by BMI. However, the tool covers most aspects of the recently published GLIM criteria for malnutrition [10]. Thirdly, selection bias is likely, as the website www.goedgevoedouderworden.nl focuses on participants that are interested in undernutrition and healthy food for older adults. Thus, prevalence rates of undernutrition and nutritional risk factors cannot be generalized towards the Dutch population. Further, as with online (self-)screening, this method has its limitations: Participants could have filled in questionnaires more than once, resulting in biased results. Further, data filled in by an informal care giver could have been biased. Finally, no information is available on important confounders such as marital status, health status, health literacy, and physical activity.

## 5. Conclusions

In conclusion: Based on the self-tests filled in at www.goedgevoedouderworden.nl—translated as “Healthy eating for healthy aging”—over half of the older participants were at risk of undernutrition, and four out of five reported early determinants of nutritional risk. Risks increased with increasing age. Self-testing of undernutrition may be a useful, contemporary, and easy, accessible way to reach older adults who are at nutritional risk and may thus contribute to early identification and prevention of undernutrition. 

## Figures and Tables

**Table 1:** Mean scores on SCREEN II and proportions <54 point.

Age	Total Score	Mean Difference
Total (*n* = 2470)	46.1 (SD:8.3)	
Age: 65–74 (*n* = 1428)	47.8 (SD:7.1)	Reference category
75–84 (*n* = 802)	45.1 (SD:8.7)	**−2.7 [95%CI: −3.3; −2.0]**
≥85 (*n* = 240)	39.2 (SD: 9.0)	**−8.6 [95%CI: −9.6; −7.5]**
	Proportion <54	Odds ratio
Total (*n* = 2470)	2078 (84.1%)	
Age: 65–74 (*n* = 1428)	1162 (81.4%)	Reference category
75–84 (*n* = 802)	686 (85.5%)	**1.4 [95%CI: 1.1; 1.7]**
≥85 (*n* = 240)	230 (95.8%)	**5.3 [95%CI: 2.8; 10.1]**

Continues data are shown as mean (difference) with standard deviation or 95%CI. Categorical data are shown as number with percentage or odds ratio’s with 95%CI.

**Table 2:** Nutritional risk factors according to the 17 questions of SCREEN II, compared between age categories.

Nutritional Risk Factor	65–74 Years n = 1428	75–84 Years n = 802	≥85 Years n = 240	Total n = 2470	*p*-Value **
**Change in weight last six months**					**<0.001**
Gained or lost ≥ 2.5kg *	509 (35.6%)	301 (37.5%)	145 (60.4%)	955 (38.7%)	
Remained stable	919 (64.4%)	501 (62.5%)	95 (39.6%)	1515 (61.3%)	
**Unintentional weight last six months**					**<0.001**
Yes *	101 (7.1%)	88 (11,0%)	52 (21.7%)	241 (9.8%)	
No	1327 (92.9%)	714 (89.0%)	188 (78.3%)	2229 (90.2%)	
**Perception body weight**					**<0.001**
More or less than it should be *	936 (65.5%)	479 (59.7%)	131 (54.6%)	1546 (62.6%)	
Just right	492 (34.5%)	323 (40.3%)	109 (45.4%)	924 (37.4%)	
**Limitation or avoiding certain products**					**0.003**
Limitation and avoiding *	581 (40.7%)	303 (37.8%)	120 (50.0%)	1004 (40.6%)	
No limitation or avoiding	847 (59.3%)	499 (62.2%)	120 (50.0%)	1466 (59.4%)	
**Skipping meals**					**<0.001**
Rarely or never	1180 (82.6%)	619 (77.2%)	139 (57.9%)	1938 (78.5%)	
Sometimes or more frequent *	248 (17.4%)	183 (22.8%)	101 (42.1%)	532 (21.5%)	
**Appetite**					**<0.001**
(Very) good	1202 (84.2%)	572 (71.3%)	101 (42.1%)	1875 (75.9%)	
Fair or poor *	226 (15.8%)	230 (28.7%)	139 (57.9%)	595 (24.1%)	
**Portions of fruit or vegetables per day**					**<0.001**
Four or more	532 (37.3%)	230 (28.7%)	40 (16.7%)	802 (32.5%)	
Three or less *	896 (62.7%)	572 (71.3%)	200 (83.3%)	1668 (67.5%)	
**Eating meat, eggs, fish or meat substitute**					**<0.001**
Once a day or less *	755 (52.9%)	453 (56.5%)	161 (67.1%)	1369 (55.4%)	
More than once a day	673 (47.1%)	349 (43.5%)	79 (32.9%)	1101 (44.6%)	
**Dairy products per day**					**0.011**
Once a day or less *	805 (56.4%)	414 (51.6%)	148 (61.7%)	1367 (55.3%)	
More than once a day	623 (43.6%)	388 (48.4%)	92 (38.3%)	1103 (44.7%)	
**Fluid use per day**					**<0.001**
≤ four glasses *	290 (20.3%)	227 (28.3%)	99 (41.3%)	616 (24.9%)	
≥ five glasses	1138 (79.7%)	575 (71.7%)	141 (58.8%)	1854 (75.1%)	
**Problems with biting or chewing**					**<0.001**
Sometimes or often *	131 (9.2%)	138 (17.2%)	98 (40.8%)	367 (14.9%)	
Rarely or never	1297 (90.8%)	663 (82.8%)	142 (59.2%)	2102 (85.1%)	
**Problems with coughing, choking or pain when swallowing**					**<0.001**
Sometimes or often *	207 (14.5%)	159 (19.8%)	67 (27.9%)	433 (17.5%)	
Rarely or never	1221 (85.5%)	643 (80.2%)	173 (72.1%)	2037 (82.5%)	
**Eating meals together**					**<0.001**
Sometimes or fewer *	457 (32.0%)	406 (50.6%)	142 (59.2%)	1005 (40.7%)	
Regularly/nearly always	971 (68.0%)	396 (49.4%)	98 (40.8%)	1465 (59.3%)	
**Meal preparation**					**<0.001**
Meal preparation is hard/I do not enjoy the meals that were prepared for me *	508 (35.6%)	356 (44.4%)	112 (46.7%)	976 (39.5%)	
Enjoy preparing meals/I enjoy the meals that were prepared for me	920 (64.4%)	446 (55.6%)	128 (53.3%)	1494 (60.5%)	
**Use of meal replacements/supplements**					**<0.001**
Sometimes or more frequent *	111 (7.8%)	83 (10.3%)	38 (15.8%)	232 (9.4%)	
Rarely or never	1317 (92.2%)	719 (89.7%)	202 (84.2%)	2238 (90.6%)	
**Problems with doing groceries**					**<0.001**
Rarely or never	1301 (91.1%)	604 (75.3%)	134 (55.8%)	2039 (82.6%)	
Sometimes or more often *	127 (8.9%)	198 (24.7%)	106 (44.2%)	431 (17.4%)	

Note: Data are presented as number (percentage), * Risk factors according to Keller et al. [14], ** *p*-value based on the chi square test.

**Table 3:** Proportions ‘Short Nutritional Assessment Questionnaire for 65+’ (SNAQ) score and odds ratio’s compared between age categories.

Age	Low Risk	Moderate	High	Odds Ratio Moderate/High vs. Low	Odds Ratio High vs. Low/Moderate
Total (*n* = 687)	256 (37.3%)	41 (6.0%)	390 (56.8%)		
Age:					
65–74 (*n* = 278)	139 (50%)	17 (6.1%)	122 (43.9%)	Reference category	Reference category
75–84 (*n* = 240)	82 (34.2%)	8 (3.3%)	150 (62.5%)	**1.9 [95%CI: 1.4; 2.8]**	**2.1 [95%CI: 1.5; 3.0]**
≥85 (*n* = 169)	35 (20.7%)	16 (9.5%)	118 (69.8%)	**3.8 [95%CI: 2.5; 6.0]**	**3.0 [95%CI: 2.0; 4.4]**

Data are shown as number (percentage) or as odds ratio with 95% CI.

**Table 4:** Risk factors of adjusted SNAQ^65+^, compared by age-group.

Age	Weight Loss > 4 kg	Problems Appetite	Problems Walking Stairs	Low (Age-Specific) BMI
Total (*n* = 687 / 460) *	234 (34.1%)	151 (33.4%)	143 (31.6%)	156 (34.4%)
65–74 (*n* = 278 / 211) *	67 (24.1%)	54 (25.6%)	44 (20.9%)	55 (26.1%)
75–84 (*n* = 240 / 155) *	90 (37.5%)	50 (33.3%)	50 (33.3%)	63 (40.6%)
≥85 (*n* = 169 / 93) *	77 (45.6%)	47 (51.6%)	49 (53.3%)	41 (43.6%)

Data are shown as number (percentage). * First number of participants only applies to weight loss, second number to ‘problems appetite’, ‘problems walking stairs’, and ‘BMI < 20 kg/m2’.
